# Obstinate Vomiting in Pregnancy

**Published:** 1881-06

**Authors:** 


### OBSTINATE VOMITING IN PREGNANCY.

Bailly has recently (*Archives de Tocologie*, January, 1881,) found good results from the application of a blister to the epigastrium, and ice along the spine in cases of obstinate vomiting from pregnancy. The remedy is rather heroic and, if used at all, should be used only as a last resort in cases where abortion is threatened, and which fail to yield to milder treatment.

